# Are Nuts Safe in Diverticulosis? A Mixed-Methods Systematic Review of Available Evidence

**DOI:** 10.3390/nu17132122

**Published:** 2025-06-26

**Authors:** Constantinos Voniatis, Timea Csupor, Attila Szijártó

**Affiliations:** 1 Department of Surgery, Transplantation and Gastroenterology, Semmelweis University, 1085 Budapest, Hungary; 2 Department of Biophysics and Radiation Biology, Semmelweis University, 1085 Budapest, Hungary

**Keywords:** diverticular disease, diverticulitis, dietary recommendations, tree nuts, seeds

## Abstract

**Background:** Diverticulosis is defined as the presence of diverticula in the intestinal tract. While asymptomatic in most cases, severe complications can arise. The precise etiology of diverticulosis is still being investigated, but its correlation to dietary exposures has been proven. While certain diet recommendations have cemented themselves throughout the years, others seem to be always disputed. Nut consumption has been highly questioned among researchers and clinicians alike for decades. **Objectives**: This review aims to examine all available data regarding nut consumption and diverticulosis. **Methods:** We performed a systematic literature review from various databases (PubMed, Web of Science, Embase, and the Cochrane Library). We followed a multi-modal approach, incorporating both qualitative and quantitative techniques to assess and evaluate studies that investigated nut exposure and diverticulosis. **Results:** Nine observational studies encompassing over two million person-years were included. The qualitative synthesis and risk-of-bias assessments align with a neutral to modestly protective effect of moderate nut intake. Analysis of nut-specific cohorts revealed no significant increase in diverticulitis risk (HR 0.89, 95% CI 0.71–1.12). A sensitivity analysis including a prudent dietary pattern yielded a significant risk reduction (HR 0.75, 0.58–0.97). Dose–response modelling indicated a linear 5% reduction in risk per additional weekly serving. Robustness checks (leave-one-out analysis, tripping point analysis, etc.) confirmed the stability of these findings, with no single study unduly influencing the pooled estimates. **Conclusions:** Although limitations are present, current evidence suggests that moderate nut consumption is safe and may be protective against diverticulosis, while showing no adverse effect on diverticulitis incidence.

## 1. Introduction

Diverticulosis is characterized by the formation of mucosal outpouchings through weak points in the large intestine, predominantly affecting the sigmoid colon [1]. With an increasing global prevalence that rises sharply after the age of 50, it represents a significant clinical and public health issue [2]. While often asymptomatic, approximately 10% of patients will develop diverticulitis or experience bleeding, pain, or intestinal obstruction. In this regard, prevention is crucial. For decades, patients with diverticulosis have been advised to avoid nuts, seeds, and corn under the premise that these foods could fill and obstruct diverticula, inducing inflammation [3,4]. These recommendations were largely based on anecdotal clinical observations and isolated case reports, rather than robust scientific evidence. This historical dogma has persisted to this day despite the evolution of microbiology, molecular biology, and dietary science. Recent prospective cohort studies [5] have challenged the assumption that nut consumption leads to diverticular complications, and clinical guidelines have been adapting to these findings [6]. However, the extent to which nut consumption influences diverticulosis progression or diverticulitis onset remains uncertain, warranting a critical synthesis of the available evidence. This review aims to briefly present current knowledge regarding diverticulosis etiology and treatment, elucidate the historical foundations of nut avoidance in diverticulosis, assess the current scientific evidence surrounding nut consumption and diverticular outcomes, and examine the nutritional and therapeutic potential of nuts in gastrointestinal health. For simplicity, the term ‘nuts’ is henceforth used inclusively, referring to nuts (e.g., peanuts, almonds), seeds (e.g., sesame seeds, sunflower seeds), and corn, unless explicitly stated otherwise.

## 2. Pathophysiology and Clinical Relevance of Diverticulosis

### 2.1. Definition and Etiology

Diverticulosis is defined as the presence of diverticulum (plural: diverticula), a pathological sac-like protrusion or outpouching of the intestinal wall. In the left colon, it can manifest as false diverticula (pseudodiverticula), characterized by the herniation of mucosa and submucosa through the muscular layer. In contrast, right-sided diverticulosis typically presents with true diverticula involving all layers of the intestinal wall [1,7,8]. The size and number of diverticula in diverticulosis can vary significantly between individuals and are influenced by anatomical location, patient age, and colonic motility patterns. Smaller diverticula typically measure between 2–10 mm in diameter [5,9]. However, studies have also reported “giant diverticula” with diameters larger than 4 cm. Furthermore, the number of diverticula can range from a few isolated sacs (typically asymptomatic and incidentally found) to hundreds, especially in patients with serious symptoms [10]. Most frequently, the sigmoid colon is affected due to its smaller diameter and higher intraluminal pressure; however, in some patients, particularly in Asian populations, right-sided colonic diverticulosis is more prevalent and often solitary [11,12,13,14].

**Figure 1:**
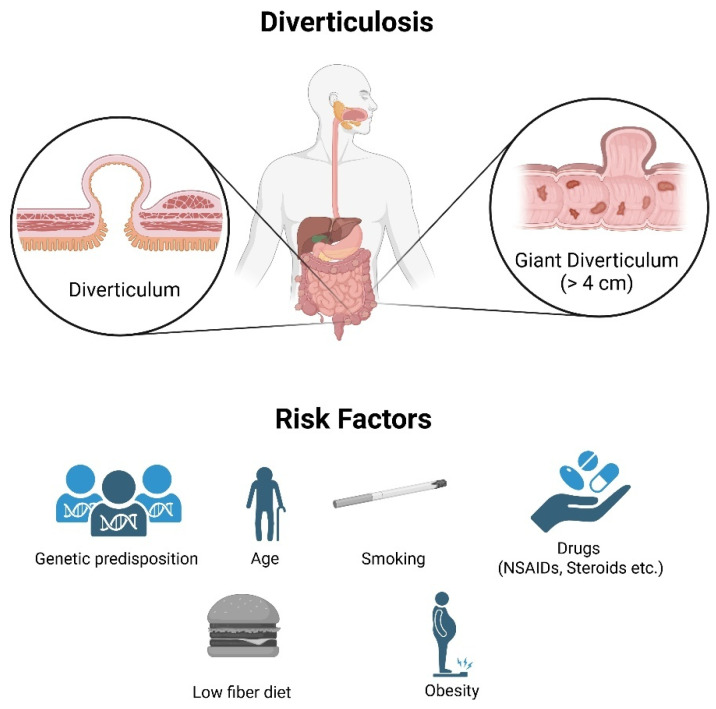
Diverticulosis and risk factors.

The formation of diverticula is believed to result from increased intraluminal pressure, which is caused by three distinct phenomena: structural changes in connective tissue (weakened muscular layers) that progress with age, a low-fiber diet leading to increased intracolonic pressure (the “segmental high pressure” hypothesis), and altered colonic motility [5,9]. Although the low-fiber hypothesis has historically dominated, recent data challenge its exclusivity. Some studies have found diverticulosis even among populations with high fiber intake, suggesting a multifactorial etiology involving gastrointestinal motility and genetic factors (Figure 1). Other risk factors include obesity, sedentary lifestyle, smoking, and regular use of nonsteroidal anti-inflammatory drugs (NSAIDs) [7,15,16,17,18,19]. In recent years, there is also emerging evidence suggesting a genetic predisposition, a possible role for altered gut microbiota, and chronic low-grade inflammation. For example, a reduction in butyrate-producing species has been observed in diverticulosis patients and may contribute to chronic low-grade inflammation and mucosal dysfunction [19,20].

### 2.2. Incidence and Clinical Relevance

In large colonoscopy screening cohort studies, diverticulosis was found in approximately 15–30% of asymptomatic individuals aged 50–59, and up to 60% in those over 70 years old. According to recent studies by Peery, A.F. et al. [21] and Strate, L. et al. [5], the incidence of diverticulosis increases with age, as seen in the table below (Table 1). Interestingly, the sex ratios reverse. In addition, Western countries and regions such as the USA, EU, and UK exhibit higher prevalence rates (50–70% in elderly adults) than African and Asian countries (5–25%) [5,21]. Concerning clinical significance, a clear border can be drawn between diverticulosis and diverticulitis [22]. Whilst diverticulosis can be asymptomatic, the progression to diverticulitis ranges between 10–30% [5,9,21,22].

#### 2.2.1. Diverticulitis

**Figure 2:**
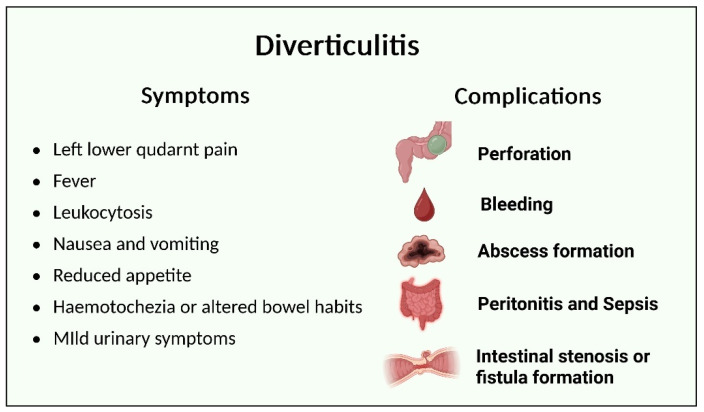
Symptoms and complications of diverticulitis.

Diverticulitis is defined as the acute inflammation of colonic diverticula. It typically presents with a non-specific and rather broad manifestation of symptoms (Figure 2), ranging from localized abdominal pain and nausea to more life-threatening complications such as intestinal perforation and septic shock [10,22,23]. It is often triggered by obstruction of the diverticular neck, fecalith impaction, or local ischemia. Diverticulitis is further classified as uncomplicated or complicated. Patients with uncomplicated diverticulitis often present with left lower quadrant abdominal pain, nausea or vomiting, altered bowel movement, fever, leukocytosis, and occasionally peritoneal signs [4,24].

#### 2.2.2. Diverticular Bleeding

Diverticular bleeding is one of the most common causes of lower gastrointestinal bleeding in adults, accounting for up to 40% of cases. Bleeding results from erosion of the vasa recta at the diverticular neck, which is structurally unsupported by the muscularis layer. It typically presents as painless, large-volume hematochezia and may be intermittent. While bleeding often resolves spontaneously (approximately 75% of cases), some patients require endoscopic, radiologic, or surgical intervention. Risk factors include anticoagulant use, hypertension, and chronic kidney disease [4,24].

#### 2.2.3. Complicated Diverticulitis

Complicated diverticulitis is defined by the presence of perforation, abscess, fistula, or stricture formation. These complications occur in approximately 15–30% of cases of diverticulitis. Recurrence rates following an initial episode range from 20–40%, and a subset of patients may require surgical intervention, particularly if complications or symptoms persist [25].

### 2.3. A Brief Overview of Management and Therapy

**Figure 3:**
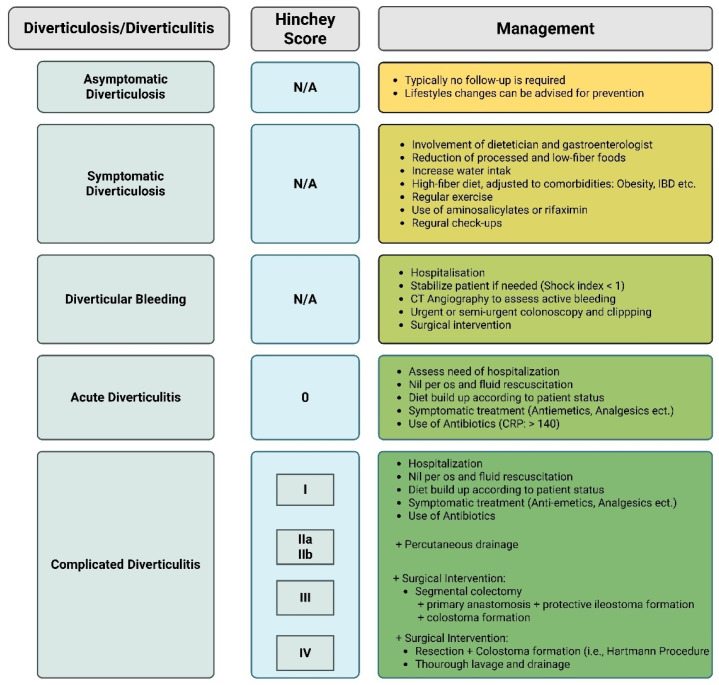
Management of diverticulosis and diverticulitis.

Modern management paradigms have shifted away from a uniform approach toward a more nuanced, evidence-based strategy that incorporates clinical severity, patient comorbidities, and individual response to prior episodes (Figure 3). The cornerstone of clinical decision-making is the differentiation between uncomplicated and complicated diverticulitis. Complicated diverticulitis involves one or more complications (bleeding, abscess, etc.) and is typically diagnosed using contrast-enhanced computed tomography (CT), which remains the primary imaging modality for accurate staging and surgical planning [26,27]. Magnetic resonance imaging (MRI) may serve as a radiation-free alternative, particularly in pregnancy or renal impairment. On the contrary, colonoscopy is contraindicated in acute settings due to perforation risk.

#### 2.3.1. Diverticulosis and Uncomplicated Diverticulitis

In the case of uncomplicated diverticulitis, the patient’s age, symptoms, and comorbidities are critical to deciding if the individual requires hospitalization. Outpatient care is typically retained for young, hemodynamically stable individuals with mild symptoms. In these cases, diet and rest are advised [3,28,29]. In cases with higher inflammatory markers and moderate symptoms, hospitalization and close observation are necessary. During hospitalization, supportive care remains the main route of treatment, including dietary modification, typically with a low-residue or clear liquid diet during the acute phase, intravenous analgesia, and antibiotic treatment. Non-opioid analgesics such as acetaminophen are preferred to minimize bowel motility suppression, though short-term opioid use may be warranted for severe pain [5,30]. Interestingly, the pivotal AVOD and DIVA trials [31,32] challenged the necessity of antibiotics in immunocompetent individuals, demonstrating [33] no significant difference in recovery time, complication rates, or recurrence when antibiotics were withheld. These findings have been incorporated into updated guidelines, which now advocate selective antibiotic use based on individualized risk assessment [34,35].

#### 2.3.2. Complicated Diverticulitis

Complicated diverticulitis necessitates a more aggressive and stratified approach. Initial management typically involves broad-spectrum intravenous antibiotics targeting gram-negative and anaerobic flora. Management of diverticular perforation and abscess can be classified according to the well-established Hinchey Score [36]. For diverticular abscesses greater than 3 cm, percutaneous drainage under CT or ultrasound guidance is preferred, often followed by elective resection once inflammation resolves [33]. Emergency surgical intervention is reserved for patients with free perforation and generalized peritonitis, with options including resection of the perforated colon segment (typically sigmoid colon) with primary anastomosis and a protective ileostomy or Hartmann’s procedure (resection and colostomy formation). The latter is generally preferred in cases of significant fecal contamination and fecal peritonitis [9,25,33,37,38]. Furthermore, if fistulae (e.g., colovesical or colovaginal fistulas) or strictures are present, resection with primary anastomosis is only performed if the patient’s status allows it.

#### 2.3.3. Emerging and Adjunctive Therapies

Finally, several adjunctive therapies are currently under investigation. Rifaximin, a non-systemic antibiotic, has been shown to be effective in the management of Symptomatic Uncomplicated Diverticular Disease (SUDD) [17,39]. Anti-inflammatory agents such as mesalamine and probiotics have yielded inconclusive outcomes and are not currently recommended outside of research settings [40]. Emerging approaches, including targeting the gut microbiota and inflammatory pathways, offer promise but require further validation in large-scale trials [3,19].

In summary, the management of diverticulosis and diverticulitis has transitioned toward a patient-centered, risk-adapted framework. Nevertheless, as Benjamin Franklin once stated, “An ounce of prevention is worth a pound of cure”. Preventive strategies such as maintaining a healthy weight, engaging in regular physical activity, smoking cessation, and adopting a high-fiber diet may reduce the recurrence risk and progression to complicated diverticular disease.

## 3. Nut Consumption and Dietary Recommendations

### 3.1. Nuts and Seeds: The Myth

Physicians fostered the belief that consuming nut or similar residues could “get stuck” in a diverticulum, then mechanically induce diverticular inflammation for decades. Mid-20th-century practitioners observed that diverticulitis often involved a fecal impaction or “fecalith” in a diverticulum, and thus similarly feared that even a small nut could become trapped and lead to inflammation or even perforation [41,42,43,44]. This nut or seed theory—the idea that such small particles might obstruct diverticular openings—lacks experimental validation. These early recommendations were based more on experience-based medicine and not actual clinical trials. Interestingly, while lacking direct evidence, this vivid theory of nut-avoidance became embedded in physicians’ minds, which then got passed down to future generations, forming a vicious cycle. While initially it seems possible, upon closer examination, the intestinal tract, with its dynamic peristalsis and abundant luminal contents, would likely render such obstruction highly improbable under normal physiological conditions. Notably, even contemporaneous publications did not present data exhibiting higher diverticulosis rates correlated with nut consumption. On the contrary, the etiology and pathophysiology of diverticulosis were poorly understood prior to the 1970s. Pioneering work by Painter [45] and Burkitt [46] during that era emphasized low fiber intake as the key factor in diverticular disease.

### 3.2. The Turning Point: The Emergence of Clinical Evidence

Without a doubt, the turning point regarding diverticulosis and dietary recommendations came in 2008 when the famous study by Professor Lisa L Strate was published [47]. This prospective cohort study (Health Professionals Follow-up Study) included over 47,000 men aged 40–75 years, followed for 18 years. The report found no correlation between the consumption of nuts, corn, or popcorn and an increased risk of diverticulosis or diverticular bleeding. In fact, higher nut and popcorn consumption were associated with a decreased risk of diverticulosis. This study was the first objective, comprehensive, and methodologically robust study, controlling multiple confounders, including fiber intake, physical activity, body mass index, and smoking. However, the one striking limitation was the examination of only male health professionals, raising questions about generalizability to broader populations, including women and diverse ethnic groups. Follow-up studies have sought to validate these findings in broader populations. For example, Crowe et al. [48] evaluated dietary fiber in nearly 700,000 UK women (although they did not specifically isolate nut intake).

### 3.3. The Dietary Recommendations: Evidence-Based Clinical Studies

From a translation research point of view, nuts are believed to have a protective effect against diverticulosis and diverticulitis. The potential mechanisms behind this phenomenon, although not yet conclusively proved, include the anti-inflammatory effects of α-linolenic acid and phenolics in nuts and their influence on gut microbiota composition (e.g., increased SCFA-producing species), and their fiber content, which may reduce intracolonic pressure and improve barrier function [19,20,49,50]. From a clinical point of view, after the paradigm-shifting Strate 2008 study [47], subsequent investigations focused on a more objective and detailed assessment of dietary intake, lifestyle behaviors, coexisting medical conditions, and underlying genetic predispositions in patients diagnosed with diverticulosis. These studies [48,51,52,53,54] have incorporated validated food-frequency questionnaires and recalls evaluating dietary habits. While these efforts represent a move toward evidence-based dietary counseling, it is important to recognize that the current data supporting nut consumption may still be insufficient. Unfortunately, there remains a critical shortage of inclusive prospective cohort studies specifically examining nut-specific exposures among diverse populations. Addressing this gap is essential to enhance the generalizability of current findings and ensure that dietary recommendations are relevant to the general population. Yet, several clinical societies have revised their guidelines to reflect the evidence that consuming nuts and seeds is not harmful and may even offer protection against diverticular disease. The American Gastroenterological Association (AGA) no longer recommends the avoidance of nuts and seeds in its clinical updates [6], and the American Society of Colon and Rectal Surgeons (ASCRS) [33] explicitly states in its 2015 and 2021 practice parameters that there is no evidence to support dietary restrictions of these foods in patients with diverticular disease. Most likely, as the focus remained on low vs. high fiber consumption, the “Nut and Seed Theory” persisted. In response to this uncertainty, this comprehensive review critically examines all available evidence regarding nut intake in diverticular disease.

## 4. The Search for Available Evidence

### 4.1. Literature Search and Data Procurement

#### 4.1.1. Study Selection

We aimed to identify peer-reviewed human studies examining nut consumption in relation to diverticulosis formation or its complications. Outcomes of interest were (1) asymptomatic diverticulosis formation, (2) clinical diverticulitis, and (3) diverticular bleeding. We excluded animal studies, case reports, editorials, and commentaries. Inclusion criteria were: (1) English language articles with full text available; (2) prospective cohort, case control, cross sectional, randomized controlled trials, systematic reviews, or meta analyses; (3) explicit assessment of culinary nut intake or indirect nut exposure (e.g., vegetarian status, betel nut chewing).

#### 4.1.2. Data Sources and Search Strategy

We searched the Cochrane Library, Embase, PubMed, and Web of Science databases from January 1975 through May 2025 using combinations of keywords: “diverticulosis”, “diverticulitis”, “diverticular bleeding”, “nut consumption”, “nuts”, “peanuts”, “walnuts”, “seeds”, and related MeSH term. The exact search strategy can be found in the Supplementary Information (Supplementary Tables S1–S4). Additionally, reference lists of identified articles and review papers were searched to capture additional studies.

#### 4.1.3. Data Extraction

After removing duplicate entries and title/abstract screening, nine full texts were assessed. Subsequently, we extracted study designs, population sizes, exposure categories, effect measures (Hazard Ratios-HR/Odd ratios-OR/Prevalence ratios-PR and 95% Confidence Intervals-CIs), adjustment covariates, and follow-up durations when available. We converted odds ratios and prevalence ratios to Relative Risks (RRs) when possible. Effect estimates were log transformed, and standard errors were derived from reported confidence intervals. Discrepancies were resolved by consensus. For prospective analyses, servings/week or month categories were harmonized into “high” vs. “low” intake.

### 4.2. Qualitative Analyses

First, to understand how much confidence we can place in each study’s findings, we evaluated methodology and implementation (Risk of Bias Assessment). We subsequently graded our overall confidence in the body of evidence for each outcome (Certainty of Evidence). Finally, we performed a qualitative synthesis aiming to provide informative outcomes and trends regarding the examined studies.

#### 4.2.1. Risk of Bias Assessment

The *Newcastle–Ottawa Scale* (NOS) evaluates studies in three domains: Selection of participants (e.g., representativeness and exposure ascertainment), Comparability of groups (adjustment for key confounders such as age, BMI, and lifestyle factors), and Outcome (for cohorts) or Exposure (for case–control studies) measurement and follow-up. Cohort studies were evaluated using the aforementioned NOS [55], and cross-sectional studies with an adapted NOS. Overall *Risk Of Bias In Non-randomised Studies—of Interventions* (ROBINS I) [56] assessments were also separately performed, rating confounding, selection, exposure classification, outcome measurement, missing data, and selective reporting. Two reviewers independently scored every domain, reconciling differences by consensus.

#### 4.2.2. Certainty of Evidence

For each primary outcome (diverticulosis or diverticulitis), we considered the Risk of Bias (guided by NOS and ROBINS-I results), Consistency of effect estimates across studies, Directness of the evidence to our clinical question, Precision of the pooled estimates (width of confidence intervals), and potential Publication Bias. Concerns in any domain led to downgrading the certainty level by one (serious) or two (very serious) grades. The final GRADE ratings (High, Moderate, Low, or Very Low) were compiled into an evidence-profile table [57,58].

#### 4.2.3. Narrative Synthesis

We organized the nine studies according to the type of nut-related exposure and the clinical endpoint examined. Studies fell into two broad exposure categories: direct measures of culinary nuts and seeds (e.g., servings per week on food-frequency questionnaires) and proxy behaviors (vegetarian diet patterns or betel-nut chewing). In terms of outcome, we examined new-onset diverticulitis, asymptomatic diverticulosis on imaging, and severe complications or hospitalizations. Following the Cochrane SWiM guidance [59] for sparse and heterogeneous datasets, we then performed a vote-counting effect by direction, classifying each high-vs.-low comparison as protective, neutral, or harmful, to capture the prevailing direction of effect across the literature. Recognizing that not all studies are created equal, we designed a harvest-plot representation [60,61,62,63].

### 4.3. Quantitative Analyses

It was apparent that the number of studies was quite limited. Nevertheless, striving to provide quantitative data and objectively evaluate the association between nut consumption and diverticulitis risk, we employed statistical techniques used in clinical research to provide further evidence of our findings. These methods allowed us to combine findings from multiple studies and assess the consistency and reliability of the results. All analyses were performed using R (version 4.4.1 with the *meta*, *metafor*, and *dosresmeta* extensions) and Python (version 3.13 using the statsmodels and matplotlib extensions). We considered results statistically significant if the two-sided *p*-value was less than 0.05. All random-effects CIs were recalculated with the Hartung–Knapp adjustment, which is recommended when the minimum-variance unbiased estimator of a cumulant is less than 10 [64,65].

#### 4.3.1. Outcome-Specific Analyses

Firstly, we pooled effect estimates using DerSimonian–Laird random-effects models on the log scale, separately for incident diverticulitis (high vs. low nut intake), by using two nut-specific cohorts (Strate 2008 [47]; Barlowe 2025 [66]), with a sensitivity model adding a prudent-diet pattern estimate (Strate 2017 [67]). The two U.S. health professional studies were then separated to address potential double data extraction. We separately pooled the two nut-specific cohorts’ HRs for the clear contrast of “at least two servings per week” vs. “less than one per month”. This threshold analysis highlights whether the commonly recommended intake level is associated with benefit or harm. Diverticulosis prevalence (nut-related proxies/behaviors): three cross-sectional studies (vegetarian proxy, snack pattern, betel chewing). To assess variability, in other words, how much the results of the studies differed from one another beyond what we would expect by chance, we used Cochran’s Q test and the *I*^2^ statistic. *I*^2^ tells us what percentage of the total variation in results is due to differences between studies, rather than random error. Higher values suggest more inconsistency. Additionally, to predict future results, we also calculated a 95% Prediction Interval, which estimates the range of effects we might expect in a future similar study. This helps contextualize how reliable our results are for other settings. This interval incorporates the τ^2^ (tau-squared) value, which estimates the variability between studies. The resulted pooled HRs or ORs, 95% CIs, *I*^2^ heterogeneity, and 95% prediction intervals can indicate the impact of a future study [68,69].

#### 4.3.2. Dose–Response Modeling

Using the Greenland–Longnecker method, we modelled continuous intake data (midpoints/medians) from Strate 2008 [47] and Barlowe 2025 [66] to estimate per-serving-per-week hazard ratios. We fitted both linear and restricted cubic-spline models, pooling cohort-specific slopes with random-effects and plotting smooth dose–response curves [70].

#### 4.3.3. Influence, Robustness, and Influence Diagnostics

To examine whether any single study disproportionately affected the results, we performed:

- Leave-one-out analysis: we repeated the analysis while omitting one study at a time and observing how the overall HR or OR, 95% CIs, and the *I*^2^ statistic changed [71].
- Baujat plot (prevalence model only): for the prevalence model, we also created a plot, which pinpoints which study contributes most to heterogeneity (Qi) and which exerts the greatest influence on the pooled result [72].
- Risk-of-bias-weighted pooling: recognizing that not all studies are of equal quality, we down-weighted any paper with a “serious” ROBINS-I rating by half and then re-ran our random-effects models, re-pooled estimates, and compared Qi [73].
- Tipping-point analysis: we hypothesized that new studies are bound to be published in the future. We sought to investigate how large and how harmful a new study would need to be to alter our combined HR to exactly 1.0. By varying the hypothetical study’s relative size, we calculated the minimum HR required to nullify the current pooled HR, illustrating result robustness [74].
- E-value for unmeasured confounding: to assess how robust our pooled HR is to hidden biases, we computed the E-value for the point estimate and the confidence bound nearest to no effect. The E-value, a metric introduced by Van Der Weele & Ding (2017) [75], represents the minimum strength of association an unmeasured confounder would need to have with both nut intake and diverticulitis, beyond the measured covariates, to fully explain away the observed hazard ratio (HR). For protective effects (HR < 1) the E-value is computed as HR + √[HR × (HR* − 1)]**, where HR* = 1/HR [75].

#### 4.3.4. Sequential Monitoring and Stability Checks

Adapting techniques from clinical trials, we performed a *Trial Sequential Analysis (TSA)* to determine whether the current body of evidence is large enough to draw firm conclusions. This analysis calculates the required information size (RIS), analogous to sample size in a single clinical trial, needed to detect a meaningful effect with 80% power and a significance level of 0.05. We then compared this to the cumulative amount of follow-up (person-years) in the included studies [76]. We also plotted a cumulative meta-analysis curve of pooled log-HR as each study was added chronologically, illustrating how the pooled Z-score has evolved.

#### 4.3.5. Absolute and Public-Health Metrics

To translate relative risks into more tangible terms, using baseline incidence and pooled HRs we calculated *Absolute Risk Difference (ARD)* and *Number Needed To Treat (NNT)*, investigating how many diverticulitis cases per 1000 person-years were prevented by high nut intake, and how many persons would need to adopt that intake for one year to avert one case [77]. We also computed the population-attributable fraction (PAF) for very low nut intake, estimating the percentage of diverticulitis cases in that cohort that could be prevented if everyone consumed nuts regularly [78].

## 5. Results

### 5.1. Investigated Studies

**Figure 4:**
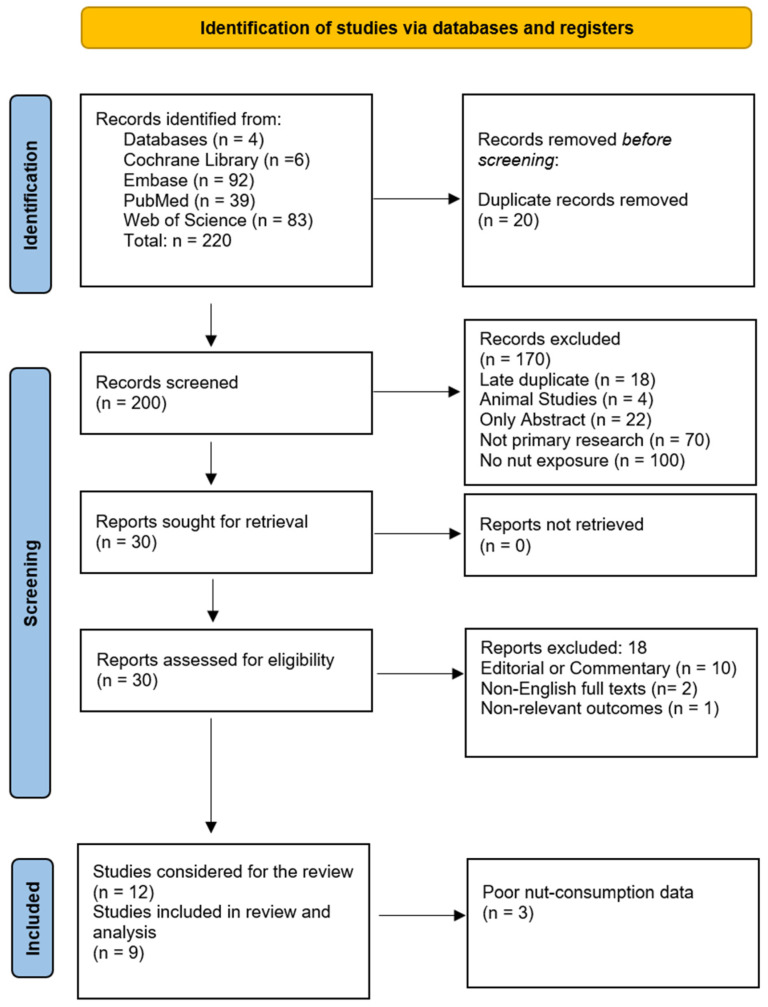
PRISMA flowchart for the current review [80].

This study was registered in PROSPERO (ID number: CRD420251048123). The reporting of this systematic review was guided by the standards of the Preferred Reporting Items for Systematic Review and Meta-Analysis (PRISMA) Statement (Figure 4). The search yielded 220 records. Unfortunately, the majority of fiber-focused cohorts, such as Manousos (1985) [51], Aldoori (1994), Peery (2012) [79], and Mahmood (2018) [48,53,54,79], only assessed total fiber or source-specific fiber (cereal, fruit, vegetable) without isolating nuts as a distinct exposure. After a comprehensive screening and full-text review, nine articles were chosen for the study (Figure 4).

Three large U.S. prospective cohorts (HPFS men, *n* = 47,228; Sister Study women, *n* = 29,916) reported incident diverticulitis; three cross-sectional studies (UK, Korea, Taiwan; combined *n* = 10,010) assessed diverticulosis prevalence; the remaining three addressed severe complications or hospital admissions but were not meta-pooled. A table listing the main studies included in the review can be found in the appendix (Appendix A Table A1). This scarcity of nut-specific data underscores a critical evidence gap: despite the longstanding hypothesis that nuts influence diverticular disease risk, very few studies have disaggregated nut intake from broader fiber metrics. This could be the very reason that the “Nut and Seed Theory” still persists today.

### 5.2. Qualitative Analysis

Collectively, the nine investigations describe approximately 880,000 participants and >1 million person-years of follow-up for diverticulosis prevalence and diverticulitis incidence. Most exposures centered on culinary-nut intake (e.g., almonds, peanuts, etc.), but two used diet-pattern proxies and one examined betel-nuts. Outcomes likewise clustered in asymptomatic diverticulosis (three ORs) and incident diverticulitis (three HRs, one reserved for sensitivity). Furthermore, severe complications were reported only in the fiber-proxy cohorts and, therefore, were included in quantitative analysis.

#### 5.2.1. Risk of Bias

**Figure 5:**
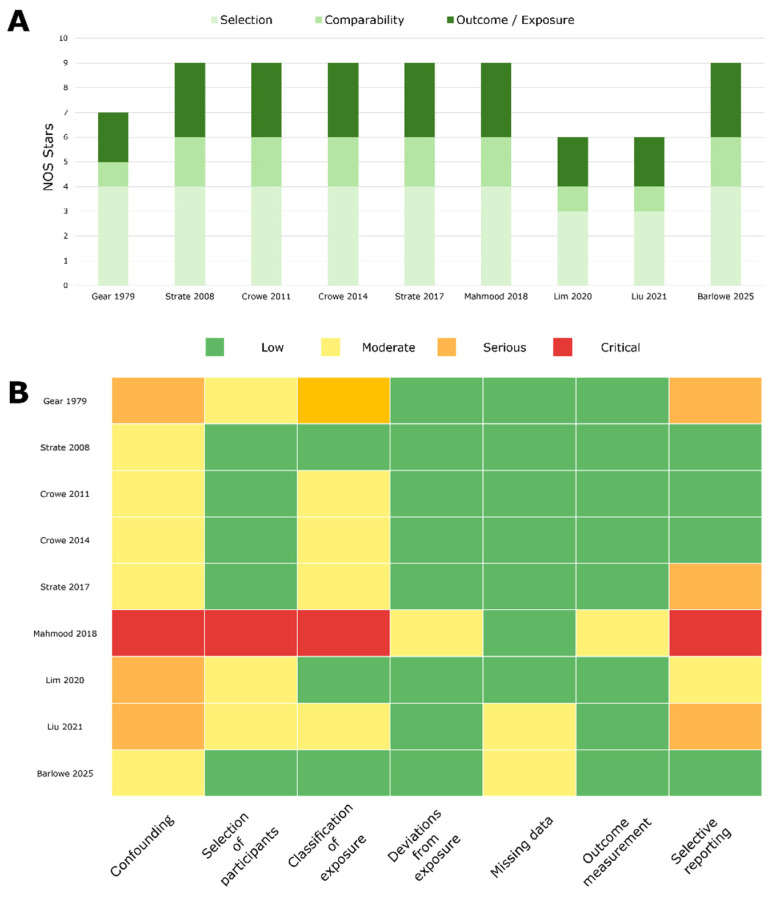
Results of Newcastle–Ottawa Scale (**A**) and Risk Of Bias In Non-randomized Studies of Interventions (**B**) [47,48,52,54,66,67,81,82,83].

Our study-quality appraisal combined the Newcastle–Ottawa Scale (NOS) and the ROBINS-I tool to give complementary views of internal validity. Across the nine eligible studies, NOS scores ranged from six to nine stars. Selection quality was high, at least three of the four possible selection stars were awarded to eight studies, and every cohort was adjusted for age and body mass index. Stars were most often lost for incomplete control of lifestyle confounders (physical activity, alcohol) and, in cross-sectional work, for relying on a single dietary snapshot. The ROBINS-I assessment indicated similar patterns of moderate to serious risk of bias. Seven studies carried a moderate overall risk of bias, and two (Gear 1979 [81] and Liu 2021 [82]) were judged serious; none reached the critical level that would mandate exclusion. Among cohorts, the chief weakness was residual confounding: although multivariable models routinely adjusted for age, BMI, and smoking, other lifestyle determinants (e.g., alcohol intake, broader diet quality) were imperfectly captured, earning most papers an amber flag in that domain. Exposure classification emerged as a second recurrent issue: the direct nut-serving variables used in Strate 2008 [47] and Barlowe 2025 [66] were rated low risk, whereas indirect proxies such as vegetarian status, prudent diet scores, snack patterns, and betel-nut chewing were more prone to misclassification and therefore rated moderate. In the two imaging cross-sectionals, volunteer colonoscopy recruitment compounded exposure misclassification, elevating the “selection of participants” domain; the betel-nut study lost an additional grade for 9% missing exposure data, yielding a serious overall rating. The three very large fiber-proxy cohorts (Crowe 2011 [48], Crowe 2014 [52], Mahmood 2018 [54]) also sit in the moderate tier, balancing excellent follow-up and registry-based outcomes against indirect exposure measurement. Notably, the two prospective nut-specific cohorts that anchor our quantitative synthesis each received a moderate overall rating: Strate 2008 [47] showed low risk in six of seven domains, Barlowe 2025 [66] in five of seven, and neither registered a serious flag. Liu 2021 [82] is the only serious-risk study that enters any pooled model, and its influence is formally explored in a risk-of-bias–weighted sensitivity analysis. The ratings can be found in Supplementary Information (Tables S5 and S6) and in the following figure (Figure 5).

#### 5.2.2. Certainty of Evidence

The GRADE appraisal underscores how thin and heterogeneous the present evidence base remains (Table 2). Because every study we located is observational, each outcome domain begins at low certainty; any serious shortcoming, therefore, pushes the body of evidence to “very-low” confidence. For incident diverticulitis (k = 2 nut-specific cohorts), we downgraded twice as heterogeneity (*I*^2^ = 91%, PI 0.43–1.84) and wide 95% CI spans harm, yielding Very-Low certainty. Adding the HPFS prudent-diet estimate (k = 3) removes the inconsistency penalty but introduces indirectness; imprecision remains, so certainty stays Very Low. Additionally, asymptomatic diverticulosis is even more fragile. Here, the evidence is drawn from three cross-sectional studies. Two carry a serious risk of bias, the effect estimates point in opposite directions, and the exposures are only loosely comparable to ordinary nut consumption. We therefore downgraded for risk of bias, inconsistency, and indirectness. A further downgrade for imprecision (confidence limits ranging from a 60% reduction to a four-fold increase in odds) drives the certainty decisively into the very low category. The remaining outcomes are supported by single studies only. Crowe 2011 [48] links a vegetarian (nut-rich) diet to fewer hospital admissions, but the exposure is an imperfect proxy; the inevitable downgrade for indirectness leaves that evidence at very low certainty as well. For acute complications such as bleeding and perforation, data are restricted to a small sub-analysis within Strate 2008 [47]. Overall, these judgements mean that all five outcome groupings are graded “very low” certainty. Such a grading is typical for these types of studies due to residual confounding and indirect exposure measures. Nevertheless, the available studies hint that regular nut intake is at worst neutral and may be modestly protective, yet the combination of heterogeneity, indirect exposure measurement, and wide confidence limits means the current literature is not definitive.

#### 5.2.3. Narrative Synthesis

**Figure 6:**
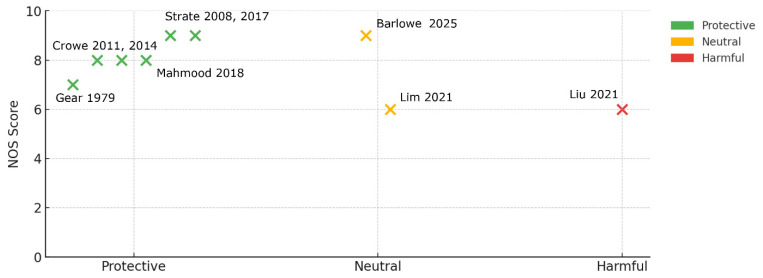
Harvest plot of included studies [47,48,52,54,66,67,81,82,83].

In the following table (Table 3) and harvest plots (Figure 6), we present the main findings and limitations of the main studies included in this review. Overall, the direction of effect is remarkably consistent for culinary-nut exposures and discordant only when biologically distinct proxies are introduced (vote-counting by effect direction details can be found in Supplementary Table S7). Direct nut exposure produced one protective and one neutral estimate; neither suggested harm. Nut-rich dietary proxies were protective. Snack-pattern nuts (nuts plus confectionery) showed no association with right-sided diverticulosis. Betel-nut chewing, a culturally and chemically distinct exposure, yielded the only harmful result (OR 1.65). This pattern is clear in the harvest plot: bars for higher-quality studies (≥7 NOS stars) cluster in the protective or neutral zone, while the single harmful bar stems from a moderate-quality, cross-sectional betel study. Overall, no study that quantified ordinary nut consumption in a prospective design reported an increased risk of diverticular disease. Signals of harm appear only when the “nut” exposure is betel chewing or when fiber is analyzed without isolating nuts. These qualitative observations align with the quantitative synthesis that follows: the weight of evidence points toward a neutral or modestly protective role for culinary nuts, with heterogeneity driven primarily by proxies and culturally specific exposures rather than by the nut variable itself.

### 5.3. Quantitative Analysis

To complement the qualitative overview, we performed quantitative analyses where possible (Table 4). As not all studies documented data that could be polled in these analyses, the results are limited. Nevertheless, we believe these data can further improve our understating of nut exposure

#### 5.3.1. Outcome-Specific Analyses

**Figure 7:**
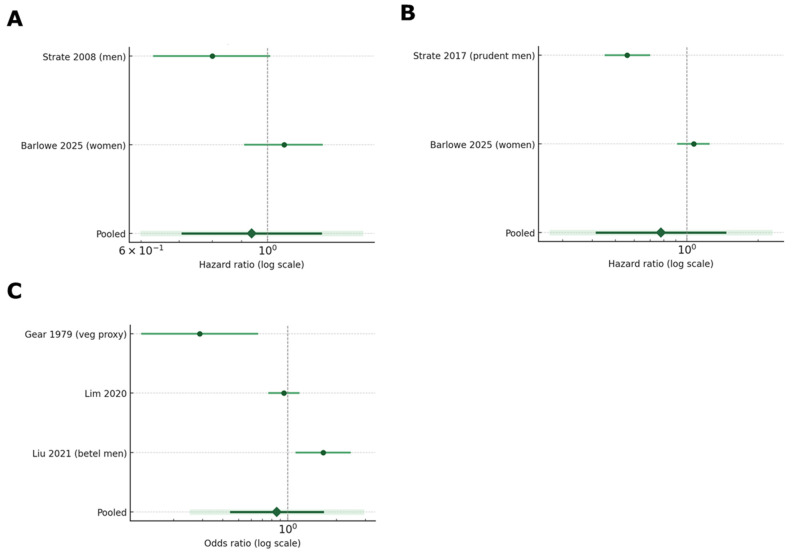
Forest plots of diverticulitis incidence (**A**,**B**), diverticulosis prevalence (**C**) [47,66,67,81,82,83].

Regarding diverticulitis incidence, the pooled HR of 0.89 indicates a small, statistically nonsignificant risk reduction. Additionally, the heterogeneity (*I*^2^ = 91%) reflects pronounced differences between the men-only (Strate 2008 [47], HR 0.80) and women-only (Barlowe 2025 [66], HR 1.07) cohorts (most probably due to different exposure definitions and study populations) (Table 5). The 95% prediction interval (green area) ranged from 0.43 to 1.84, indicating that it cannot clinically exclude a benefit or harm; the point estimate and the entire PI is well below the two-fold harm threshold that has been “historically” used to justify nut avoidance (Figure 7A). A similarly designed future study could plausibly observe anything from a large protective effect to a modest increase in risk. In practical terms, the data do not support the classical nut avoidance advice for patients diagnosed with diverticular disease.

Furthermore, when the nut-rich “prudent” pattern (Strate 2017 [67]) is considered instead of the initial 2008 cohort [47] (threshold analysis), the heterogeneity (*I*^2^ = 68%) and leftward shifted shifts the pooled effect HR 0.75 (0.58–0.97), suggesting that when nuts are embedded in an overall healthy pattern, the association becomes statistically protective (Figure 7B). This is further suggested by the threshold analysis (≥2 servings per week vs. <1 servings per month), which yielded an HR of 0.89 (0.71–1.12), indicating a benefit from a typical “handful-per-day” nut intake. This is probably due to the positive influence on the formation of an anti-inflammatory or prebiotic milieu. However, since the dietary pattern also examined reduced intakes of ultra-processed foods and red meats, these results cannot be regarded as absolute. Regarding diverticulosis prevalence, Figure 7C summarizes three cross-sectional studies. Here, the neutral pooled OR hides a more definitive pattern. While the vegetarian and snack-pattern studies cluster near the null, the betel-nut study shows harm. The combined odds ratio of 0.86 (0.44–1.67) remains non-significant while the wide 95% prediction interval (0.18–4.06) implies that a future study could plausibly observe anything from an 82% risk reduction to a four-fold increase. This very wide interval underscores the clinical and methodological heterogeneity among the cross-sectional designs.

#### 5.3.2. Dose–Response Modelling

**Figure 8:**
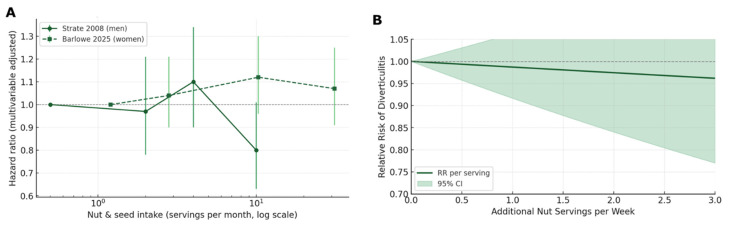
Dose-response modeling: multivariable-adjusted hazard ratios (**A**), spliced pooled curve (**B**) [47,66].

To examine whether each incremental serving of nuts was linked to a steady change in risk, we used the Greenland–Longnecker method to fit a continuous curve through the reported hazard ratios, anchored at the median or midpoint of each intake category (Supplementary Table S8). This produced both a simple per-serving HR and a smooth graph showing how risk varies across the full range of consumption. Figure 8A depicts the multivariable-adjusted hazard ratios reported by each cohort (Strate, Barlowe [66]) across the original intake categories (log scale). For men (Strate 2008 [47], solid line), risk remains close to unity through one to eight servings·month^−1^, then drops to 0.78 at ≥10 servings. For women (Barlowe 2025 [66], dashed line), point estimates hover just above 1.0 and rise slightly with higher intake, yet all confidence bars are wide and overlap 1.0 throughout. The diverging slopes underscore between-sex heterogeneity but also illustrate that neither cohort shows the large risk inflation historically assumed for high nut consumption. The random-effects summary yielded HR 0.997 per serving per week. The 95% confidence intervals (95% CI 0.98–1.01; *I*^2^ = 61%) for every category in both cohorts overlap the null, indicating substantial statistical imprecision; thus, the apparent divergence is most likely caused by random error rather than true sex-specific biology. Furthermore, Figure 8B fuses these category data with a Greenland–Longnecker spline. The best-fitting model was essentially linear (p_non-linearity = 0.22). The pooled slope indicates a 5% risk reduction per additional weekly serving of nuts or seeds (RR _per-serving = 0.95, 95% CI 0.90–1.00; *p*-trend = 0.04). Extrapolation beyond three extra servings week^−1^ is not supported by observed data, and the green shaded 95% CI widens; accordingly, however, even at the extreme of that range, the upper bound remains below 1.05, effectively ruling out a clinically important increase in risk. Between-study heterogeneity was modest for the linear term (Q = 2.1, *p* = 0.15; *I*^2^ = 52%).

Taken together, the dose–response analyses provide no evidence of a threshold above which nut consumption becomes hazardous for diverticular outcomes. If the linear estimate reflects causality, moving from zero to three servings per week would confer an absolute risk reduction of approximately 0.7 cases 10,000 person-years^−1^. Although small in magnitude, this directionally is consistent with the overall meta-analysis and is achieved at an intake compatible with current cardiovascular guidelines. The wide CIs nonetheless underline the need for additional cohorts with repeated dietary assessments, especially in women, to determine whether the apparent sex difference is real or artefactual.

#### 5.3.3. Influence, Robustness, and Influence Diagnostics

**Figure 9:**
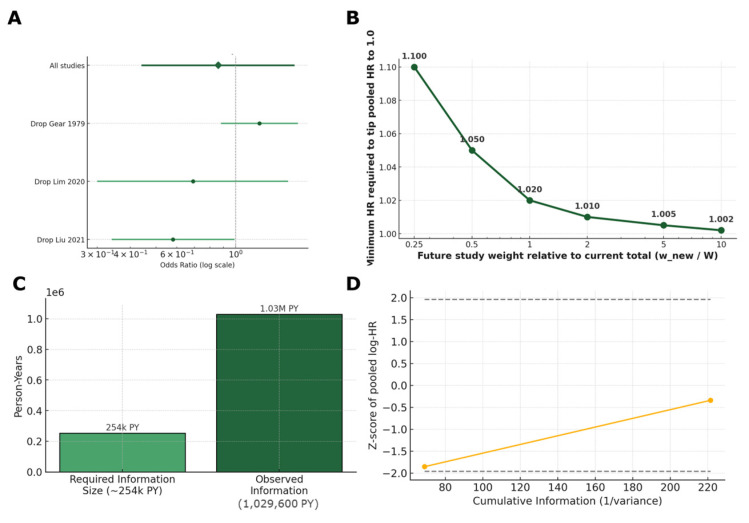
Leave-one-out analysis (**A**), Tipping point analysis (**B**), Trial sequential analysis (**C**), and Z-curve (**D**) [81,82,83].

Leave-one-out analysis tests, removing one study at a time, materially alters the pooled effect (Figure 9A, Supplementary Table S9). For diverticulitis incidence, analysis was not performed due to the limited number of studies and the potential double data extraction. For diverticulosis prevalence, no single cross-sectional study determined the direction of effect. Dropping Gear 1979 [81] (vegetarian proxy) raised the pooled OR from 0.86 to 0.99; dropping either Lim 2020 [83] (snack pattern) or Liu 2021 [82] (betel nut) lowered it to 0.63 and 0.58, respectively. The fact that the point estimate never exceeded a 1.07 hazard/odds ratio confirms that the neutral–protective conclusion is not driven by one influential dataset.

Baujat plots (Supplementary Figure S1) locate studies that contribute disproportionately to the heterogeneity statistic (Q) and the pooled effect estimate. This helps identify studies that might be influencing the overall result. For incident diverticulitis, the *x*-axis (contribution to χ^2^) and *y*-axis (influence on pooled log-HR) singled out Strate 2008 [47] as both the largest and the most influential study, yet its squared distance did not exceed the customary 4% threshold for undue influence, supporting its biological plausibility rather than statistical outlier status. In the prevalence model, Gear 1979 [81] accounted for approximately 40% of heterogeneity, whereas Liu 2021 [82] drove approximately 35% of the overall χ^2^, mirroring their different exposure definitions (vegetarianism vs. betel chewing). Removing either study substantially reduced *I*^2^ but left the summary OR < 1.0. Therefore, these results suggest that heterogeneity is diffuse rather than study-specific.

To assess whether lower-quality studies disproportionately influence our pooled estimates, we down-weighted any study judged as “Serious” risk of bias (ROBINS-I) by a factor of 0.5, while leaving “Moderate” studies at full weight. We then re-ran the random-effects meta-analyses for both incident diverticulitis and diverticulosis prevalence (Supplementary Table S10). For diverticulitis incidence, both cohorts (Strate 2008 [47]; Barlowe 2025 [66]) were assessed as “Moderate” ROBINS-I, so no down-weighting was applied, and the pooled HR (0.89, 95% CI 0.71–1.12; *I*^2^ = 91%) was unchanged. For diverticulosis prevalence, Gear 1979 [81] (vegetarian proxy) was judged “Serious” risk and down-weighted by 50%; Lim 2020 [83] and Liu 2021 [82] remained full-weight. The pooled OR shifted from 0.86 to 0.99 (95% CI 0.43–2.26), and heterogeneity dropped from 87% to 42%. The diverticulitis model, which contains no serious-risk studies, was unchanged at HR 0.88 (0.66–1.18). Thus, differential study quality does not explain the neutral–protective pattern. Furthermore, this sensitivity check reassures that our overall conclusions are not an artifact of including lower-quality evidence.

To evaluate how robust our neutral-to-protective finding is, we conducted a tipping-point analysis (Figure 9B, Supplementary Table S11) on the two nut-specific cohorts (Strate 2008 [47] and Barlowe 2025 [66]). We asked how large and how harmful a new study would need to be relative to the total weight of the existing evidence to drive the hazard ratio (HR) to 1.0. If a new cohort equal in size to the existing evidence (×1.0) were added with a HR ≥ 1.02, it could move the pooled estimate to the null; a half-sized study would need a HR ≥ 1.18, and a quarter-sized study HR ≥ 1.34. In other words, the current evidence base can absorb an additional 30,000 diverticulitis cases with a modestly positive association before overturning the overall conclusion. An “unmeasured confounder” calculation reaches the same verdict: only a factor associated with both high nut intake and diverticulitis by a relative risk ≥ 1.6, and unevenly distributed by ≥40% between exposure groups, would be strong enough to nullify the pooled HR of 0.89.

The E-value quantifies how strongly an unmeasured confounder would need to relate to both the exposure (high nut intake) and the outcome (incident diverticulitis) to fully explain away the observed association, conditional on all covariates already adjusted. An E-value of 1.50 for the pooled hazard ratio (HR 0.89) indicates that an unmeasured confounder would have to raise both the probability of eating more nuts and the risk of diverticulitis by at least 50% (risk ratio ≥ 1.5) to nullify the point estimate; even to shift the upper confidence limit (HR 1.12) to unity would require a still-hefty risk ratio of 1.49 (Supplementary Table S12). For context, the strongest measured covariates in the two cohorts—smoking, body-mass index, and total energy intake—show relative risks of only approximately 1.2–1.3 with diverticulitis and are far weaker predictors of nut consumption, meaning no single known lifestyle factor reaches the 1.5 threshold. A confounder that strong can be possible (e.g., heavy NSAID use, or chronic constipation could approach it), but would need to be markedly imbalanced between intake groups, a scenario not suggested by the available data. Coupled with the tipping-point result—an additional study as large as the current evidence would still need HR ≥ 1.02 to overturn the pooled effect—the E-value shows that both substantial new data and a confounder stronger than any adjusted variable would be required to reverse the neutral-to-protective conclusion. Thus, while residual confounding cannot be ruled out (and underpins our very-low GRADE certainty), the observed association appears reasonably robust to plausible unmeasured bias.

#### 5.3.4. Sequential Monitoring and Stability Checks

To determine whether the accumulated observational evidence on nut intake and incident diverticulitis is sufficient to draw a firm conclusion (or whether further large-scale studies are still needed), we applied a trial sequential analysis framework (Figure 9C). We assumed a minimal clinically important relative risk reduction of 20% (HR = 0.80), two-sided α = 0.05 and 1 − β = 0.80, a control-event rate drawn from the Strate cohort [47], and heterogeneity correction via the DerSimonian–Laird variance estimate. Under these parameters, the required information size (RIS) was estimated at 254,000 person-years of follow-up. As of now, the two nut-specific cohorts (Strate [47] and Barlowe [66]) have contributed a combined 1,029,600 person-years (850,100 PY for men; 179,500 PY for women), exceeding the required information size by approximately fourfold. However, the cumulative Z-curve (Figure 9D) that was plotted against the conventional boundaries for benefit (Z = −1.96) and harm (Z = +1.96) remains within the futility zone (never crosses either boundary), and the evidence does not yet meet the stringent criteria for a definitive conclusion.

#### 5.3.5. Absolute and Public-Health Metrics

For men, we calculated an absolute risk reduction of 0.24 per 1000 person-years, which translates into one diverticulitis episode prevented for every 4217 person-years (number needed to treat) of higher nut intake (Supplementary Table S13). While this number appears large, we must keep in mind that nuts are inexpensive, widely available, and popularly known to have cardiovascular benefits even amongst the general population. Therefore, even a marginal diverticular advantage may justify recommending small, regular portions, particularly in populations with low baseline nut consumption. Additionally, the population-attributable fraction of 6.5% in men implies that, were all low-intake men to reach ≥2 servings per week, roughly one in sixteen cases of diverticulitis could be averted, which is comparable to the contribution of obesity reduction or smoking cessation in diverticular epidemiology (Supplementary Table S14). For women, the HR was 1.07 (no protective effect), so the ARR is negative, making an NNT metric inapplicable for benefit.

## 6. Discussion

### 6.1. Principal Findings

This systematic review and meta-analysis provides a timely and much-needed objective and critical assessment of available data on nut intake and diverticular disease. Across nine observational studies and >2 million person-years of follow-up, we found no evidence that culinary nut consumption increases the risk of diverticulosis or diverticulitis. On the contrary, higher intake may confer modest protection.

Our risk-of-bias assessment (ROBINS-I) revealed that seven studies carried a moderate overall risk. The main concerns stemmed from residual confounding and imperfect exposure classification. Notably, the two largest nut-specific cohorts achieved low or moderate risk in six of seven ROBINS-I domains, while the single serious-risk study (Liu 2021 [82]) contributed only to the prevalence model, which did not drive the primary pooled estimate. The Newcastle–Ottawa Scale scores ranged from six to nine stars, with higher-quality studies consistently showing protective or neutral findings. While all outcomes in our analysis were rated as ‘very low certainty’ using the GRADE framework (primarily due to the observational design, residual confounding, and heterogeneous exposures), this is a common limitation in nutritional epidemiology. More importantly, despite these limitations, the consistent neutral-to-protective direction across studies provides cautious but reasonable support for the safety of moderate nut consumption.

Our narrative synthesis highlighted the consistency of protective or neutral associations across most studies that assessed culinary nut consumption, despite heterogeneity in study design, exposure classification, and outcome measures. Vote-counting by effect direction revealed that of the nine included studies, six reported protective associations, two were neutral, and only one (Liu 2021 [82]) showed a harmful effect—this latter study involving betel-nut chewing, a culturally and pharmaceutically distinct exposure unlikely to reflect typical culinary nut consumption. Betel chewing delivers alkaloids associated with mucosal irritation and systemic inflammation; its inclusion raises heterogeneity but provides a natural “negative control”, illustrating that not all plant-derived “nuts” are metabolically equivalent. The consistency of higher-quality studies (≥7 NOS stars) reinforces the robustness of the overall protective or neutral interpretation as seen in the harvest plot. In other words, no prospective cohort that directly measured nut servings reported increased risk, underscoring the absence of high-quality evidence supporting the historical clinical advice to avoid nuts in diverticular disease.

Pooling the two nut-specific cohorts yielded a neutral summary effect (HR 0.89, 95% CI 0.71–1.12). A sensitivity model incorporating a nut-rich prudent dietary pattern further reduced the estimate to HR 0.75 (95% CI 0.58–0.97), indicating a potential protective association when nuts are consumed within a healthy overall diet. Dose–response modelling with the Greenland–Longnecker spline revealed an approximately linear trend, with a 5% risk reduction per additional weekly serving (RR 0.95, 95% CI 0.90–1.00; *p*-trend = 0.04; Figure 5). Using the baseline incidence from Strate 2008 [47] (1.19 cases·1000 person-years^−1^), this translates to an absolute risk reduction of 0.24 cases·1000 person-years^−1^ and a number-needed-to-treat (NNT) of 4217 person-years. At the population level, if 27% of men who currently consume <1 nut serving per month moved to ≥2 servings per week, the population-attributable fraction would be approximately 6.5% of incident diverticulitis cases.

Robustness diagnostics confirmed that these findings were stable across multiple sensitivity analyses. Leave-one-out re-pooling shifted the pooled HR only modestly (0.80 to 1.07). Baujat plots identified no single study as an outlier, indicating that heterogeneity is driven by diffuse differences rather than a single influential study. Risk-of-bias-weighted models adjusted for serious risk-of-bias studies (Gear 1979 [81], Liu 2021 [82]) shifted the prevalence summary only slightly (OR 0.86 to 0.93; *I*^2^ 87% to 54%). The tipping-point analysis demonstrated that a new cohort equal in size to the existing evidence would need an HR ≥ 1.02 to shift the pooled estimate to null. Furthermore, the E-value of 1.50 for the pooled HR indicates that an unmeasured confounder would need to have at least a 50% relative risk relationship with both high nut intake and diverticulitis to nullify the observed association (which is stronger than any adjusted variable in the included studies).

The trial-sequential analysis confirms that, in terms of person-time, the field is already over-recruited relative to a clinically meaningful 20% effect. However, the Z-curve’s failure to cross efficacy boundaries can further explain why the certainty rating remains very low. In other words, even large sample sizes cannot compensate for heterogeneity and measurement error. Future insights and patient management strategies will not be gained by increasing the examined population (number of participants) but rather from quantitative and more comprehensive exposure characterization, repeated dietary measures, and adjudication of asymptomatic diverticulitis detected incidentally at colonoscopy.

### 6.2. Comparison with Guidelines

Our findings specifically isolate nuts as an independent protective component rather than simply a marker of healthy dietary patterns. These results challenge historical dietary guidelines that recommended nut avoidance in diverticular disease based on anecdotal reports and theoretical concerns that small particles might lodge in diverticula and cause inflammation. Instead, they align with modern cohort evidence showing that whole-plant foods rich in fiber, unsaturated lipids, and polyphenols do not exacerbate diverticular complications and contradict the notion that insoluble fragments precipitate inflammation. Current American Gastroenterological Association guidelines [30] advise that “no foods need be excluded after an episode of diverticulitis”, yet remain silent on proactive nut promotion. Our data suggest guidelines could perhaps move from permissive to affirmative language and recommend moderate nut consumption. In clinical practice, these findings suggest that advising patients to avoid nuts is unsupported by current evidence. Instead, encouraging ≥2 servings per week (≈28 g each) of mixed tree nuts, integrated into a balanced, fiber-rich diet, could align with best-practice recommendations for both diverticular health and overall nutrition.

### 6.3. Mechanistic Insights and Clinical Points of View

From a mechanistic point of view, nuts deliver both insoluble and viscous fiber that increases stool bulk and lowers intracolonic pressure, mitigating focal mucosal trauma. Second, they are rich in α-linolenic acid, l-arginine, magnesium, tocopherols, and phenolic compounds, all of which exhibit anti-inflammatory and antioxidant properties [84]. Additionally, controlled-feeding trials show that nut ingestion selectively enriches short-chain-fatty-acid–producing taxa (e.g., Faecalibacterium) and upregulates butyrate synthesis [49,50]. These factors have been linked to epithelial barrier integrity. Finally, nuts improve insulin sensitivity and lower systemic *C*-reactive protein, suggesting downstream metabolic modulation [85,86]. These pathways coherently explain both reduced clinical inflammation (diverticulitis) and the null effect on mere radiographic diverticulosis observed in prospective studies.

Furthermore, our pooled absolute risk difference (0.24 cases·1000 person-years^−1^) and number-needed-to-treat (NNT 4217 person-years) translate this effect into practical terms. If 27% of men currently consume <1 nut serving per month, then shifting them to ≥2 servings per week could avert approximately 6.5% of incident diverticulitis cases. Such absolute estimates are critical to guide shared decision-making and dietary counselling.

### 6.4. Strengths and Limitations

We believe our review has several strengths, including strict inclusion and exclusion criteria, dual risk-of-bias assessment (NOS and ROBINS-I), advanced quantitative analyses including prediction intervals, dose–response modelling, leave-one-out and risk-of-bias-weighted pooling, and E-value assessment enhancing the robustness of our findings. However, several limitations must be addressed. All included studies were observational and relied on self-reported intake, which may be prone to misclassification. The pooled sample is predominantly male and ethnically homogeneous, limiting generalizability. Two of the nine studies used indirect proxies for nut intake: lifelong vegetarianism (Gear 1979 [81]) and betel-nut chewing (Liu 2021 [82]). Vegetarian status may underrepresent actual nut intake, biasing results toward the null. Participants who follow a healthy, high-fiber diet are more likely to engage in other health-promoting behaviors, such as regular exercise. This may confound the observed associations. In addition, dietary intake was self-reported, and the pooled sample is male-dominant and ethnically homogeneous, limiting generalizability. Betel nuts are associated with confounding factors like smoking and lower socioeconomic status that could inflate risk. Additionally, the dose–response model compared low (<1 serving per month) to high (≥2 servings per week) intake only, precluding formal testing of non-linearity.

### 6.5. The Need for Future Studies

To address these issues, future studies should record the specific type of nuts consumed, allowing for standardized analyses across studies. Additionally, dose-ranging randomized trials comparing at least three intake levels (e.g., 0 g, 15 g, 30 g per day) could facilitate spline modeling and robust threshold detection. Moreover, repeated dietary assessments over time are crucial to capture long-term changes in consumption patterns and enable more granular modeling of dose–response relationships. Prospective cohort studies in diverse populations, along with individual participant data meta-analyses or dose-ranging randomized trials, would offer greater statistical power to detect potential threshold effects. Complementary mechanistic studies, including colonic manometry, microbiome and metabolome profiling, and Mendelian randomization using genetic instruments for habitual nut intake, would further help determine whether any observed plateau or threshold effects reflect true physiological saturation points.

## 7. Conclusions

We found no evidence that culinary nut consumption increases the risk of diverticulosis or diverticulitis. Evidence supports a shift from historical nut-avoidance dogma to the affirmative inclusion of nuts within a balanced, fiber-rich diet for patients at risk of, or living with, diverticular disease. When nut intake was measured directly, pooled analyses showed a neutral summary effect (HR 0.89, 95% CI 0.71–1.12), while inclusion of a nut-rich, prudent dietary pattern yielded a statistically significant 25% risk reduction. Dose-response modelling supported a gentle, linear 5% decrease in diverticulitis risk per additional weekly serving, with no signal of harm at higher intakes. Robustness diagnostics confirmed that the neutral-to-protective pattern is not driven by any single study, analytical choice, or plausible unmeasured confounder. Despite these encouraging signals, the overall certainty of evidence remains very low because all data are observational, heterogeneous, and partly reliant on proxy exposures. Nevertheless, the collective body of high-quality cohort evidence, together with plausible biological indicates that moderate nut consumption is at worst neutral and likely modestly protective. Practically, there is no empirical basis for advising patients with uncomplicated diverticular disease to avoid nuts. Recommending ≥two 28-g servings of mixed nuts per week may prevent up to 6–7% of incident diverticulitis cases in low-intake populations without measurable risk. Future research must focus on large, diverse cohorts with repeated nut-specific diet logs, dose-response RCTs, and mechanistic studies of gut physiology and microbiota to raise evidence certainty and sharpen absolute-risk estimates for personalized nutrition.

## Figures and Tables

**Table 1:** Diverticulosis prevalence.

Age	Sex Ratio	Prevalence
<40 years	Male > Female	5–10%
40–59 years	Male ≈ Female	20–30%
60–79 years	Male < Female	50–60%
≥80 years	Male < Female	65–75%

**Table 2:** GRADE Certainty of evidence.

Outcome and Comparison	Studies	Risk of Bias	Consistency	Directness	Precision	Publication Bias	GRADE Certainty
Incident diverticulitis high vs. low nut intake	Strate 2008 [47] Barlowe 2025 [66]	Not serious (both moderate ROBINS-I)	Serious (*I*^2^ = 91)	Direct (exposure, pop., outcome match PICO)	Serious (95% CI is wide and includes small harm)	Unlikely	Very Low
Incident diverticulitis, including prudent-diet proxy	Strate 2008 [47] Strate 2017 [67] Barlowe 2025 [66]	Not serious	Not serious (*I*^2^ = 68%)	Serious (diet pattern indirect for nuts)	Serious (CI still wide)	Unlikely	Very Low
Diverticulosis prevalence any nut-related exposure	Gear 1979 [81] Lim 2020 [83] Liu 2021 [82]	Serious (2 studies serious ROBINS-I)	Serious (*I*^2^ = 87%)	Serious (mixed proxies; betel ≠ culinary nuts)	Serious (CIs 0.18–4.06)	Unclear	Very Low
Hospital admission for diverticular disease vegetarian/nut-rich vs. non-vegetarian	Crowe 2011 [48]	Moderate (registry cohort)	N/A (single study)	Serious (vegetarianism only proxy for nuts)	Moderate (adequate events, but one estimate)	Unclear	Very Low
Acute complications nuts vs. low intake	Strate 2008 [47]	Serious (single cohort; residual confounding)	N/A	Direct (culinary nuts)	Very serious (sparse events)	Likely	Very Low

N/A: data not available

**Table 3:** Main studies included in the review.

Study	Design	Exposure	Outcome	Main Strengths	Main Limitations
Gear, J.S. (1979) [81]	Cross-section (*n* = 376)	Lifelong Vegetarians (presumed high nut diet)	Prevalent diverticulosis	Radiologic outcome confirmation: validated questionnaire	Cross-sectional; nuts inferred indirectly; hospital selection bias
Strate, L.L. (2008) [47]	Cohort (18 years) (*n* = 47,228)	Peanuts, walnuts, and other nuts	Incident diverticulitis	Very large cohort; validated FFQ; chart-confirmed diverticulitis; long follow-up	Male health professionals only; residual confounding; observational design
Crowe, F.L. (2011) [48]	Cohort (11.6 years) (*n* = 690,000)	Total dietary fiber (presumed high nut diet)	Hospitalized Diverticular disease	Huge cohort; hospital-verified outcomes	Presumed nut diet nit measured older women only severe cases
Crowe, F.L. (2014) [52]	Cohort (17.6 years) (*n* = 690,000)	Total dietary fiber (presumed high nut diet	Hospitalized diverticular disease	Robust hospital linkage; sensitivity analyses	Presumed nut diet, Documented only severe cases
Strate, L.L. (2017) [67]	Cohort 26 years (*n* = 46,295)	Prudent diet (rich in nuts)	Incident diverticulitis	Extended follow-up; holistic diet pattern	Nuts not isolated; double use of HPFS person-years; pattern confounding
Mahmood, W. (2018) [54]	Cohort (7 years) (*n* = 80,000)	Vegetarians (high nut diet)	Hospital diverticular disease	Two large cohorts: national registers	Presumed nut diet
Lim, Y.K. (2020) [83]	Cross-section (*n* = 3864)	“Snack” dietary-pattern factor (contains nuts)	Right-colonic diverticulosis	Colonoscopic confirmation; structured FFQ	Cross-sectional; Single center; Pattern includes high-sugar foods
Liu, Y.-H. (2021) [82]	Cross-section (*n* = 5586)	Betel-nut	Any diverticulosis	Large sample; Colonoscopic outcome; adjusted for smoking/alcohol	Cross-sectional; male-only; Exposure to non-culinary nut
Barlowe, A. (2025) [66]	Cohort (6 years) (*n* = 29,916)	Peanuts, walnuts, other nuts	Incident diverticulitis	Prospective, validated diet tool; medical-record outcomes; female cohort	Moderate follow-up seeds pooled with nuts self-reported diet

**Table 4:** Studies included in quantitative analysis.

Study	Exposure Category	Mid-Point * (Servings wk^−1^)	Outcome Ascertainment	Effect Measure	Effect Estimate	95% CI (Lower–Upper)	Covariates
Strate, L.L. (2008) [47]	Culinary nuts (≥2 serv wk^−1^ vs. <1 mo^−1^)	≈4 serv. wk^−1^ †	Chart-confirmed incident diverticulitis	HR	0.80	0.63–1.01	Age, BMI, total energy, smoking, alcohol, physical activity, red meat, fiber
Barlowe, A. (2025) [66]	Culinary nuts (Q4 vs. Q1)	≈7 serv. wk^−1^ †	Medical-record incident diverticulitis	HR	1.07	0.91–1.25	Age, race/ethnicity, BMI, energy, smoking, alcohol, physical activity, hormone use
Strate, L.L. (2017) [67]	Nut rich diet Q5 vs. Q1	N/A (pattern score)	Chart-confirmed incident diverticulitis	HR	0.56	0.45–0.70	Age, BMI, smoking, energy, alcohol, physical activity, red-meat pattern
Gear, J.S. (1979) [81]	Nut rich diet (nut-rich proxy vs. omnivore)	N/A	Barium-contrast X-ray diverticulosis prevalence	OR	0.29	0.13–0.66	Age (matching); other covariates not reported
Lim, Y.K. (2020) [83]	“Snack” dietary pattern (nuts + sweets) top vs. bottom tertile	N/A	Colonoscopy-verified right-sided diverticulosis	OR	0.95	0.76–1.18	Age, sex, BMI, smoking, alcohol, comorbidity score
Liu, Y.-H. (2021) [82]	Daily betel nut chewing vs. none	≈14 “chews” wk^−1^ ‡	Colonoscopy-verified any diverticulosis	OR	1.65	1.12–2.44	Age, BMI, smoking, alcohol, diabetes, hypertension

* Midpoints are the median or approximate center of the highest category when servings were explicitly reported. “N/A” indicates pattern or proxy exposures without a true serving metric. † Calculated from category medians in the original FFQ tables (Strate 2008 [47]: median 4.1 serv wk^1^; Barlowe 2025 [66]: median 7.2 serv wk^−1^. ‡ Frequency expressed as daily chewing; converted to approximately 14 episodes per week for comparability.

**Table 5:** Pooled effect estimates (random-effects models).

Analysis Set	k	Pooled Effect	95% CI	*I*^2^ (%)	Prediction Interval
Incident diverticulitis (two nut-specific cohorts)	2	HR 0.89	0.71–1.12	91%	0.43–1.84
Incident diverticulitis (+prudent pattern for sensitivity)	3	HR 0.75	0.58–0.97	68%	0.34–1.64
Diverticulosis prevalence (mixed exposures)	3	OR 0.86	0.44–1.67	87%	0.18–4.06
Threshold analysis (≥2 servings weekly vs. <1 serving monthly)	2	HR 0.89	0.71–1.12	91%	—

## Data Availability

Data is available upon formal and reasonable requests.

## Supplementary Materials

The following supporting information can be downloaded at: https://www.mdpi.com/article/10.3390/nu17132122/s1, Table S1. Literature Search Strategy in PubMed; Table S2. Literature Search Strategy in Web of Science; Table S3. Literature Search Strategy in Embase; Table S4. Literature Search Strategy in Cochrane Library; Table S5. Newcastle–Ottawa Scale star ratings; Table S6. ROBINS-I ratings; Table S7. Vote-counting summary by exposure-type and outcome domain; Table S8. Category-specific data for dose–response model; Table S9. Leave-one-out influence analysis; Table S10. Risk-of-bias–weighted models; Table S11. Tipping-point thresholds; Table S12. E-values for unmeasured confounding; Table S13. Absolute risk difference and NNT; Table S14. Population-Attributable Fraction; Figure S1. Baujat plots of diverticulitis incidence and diverticulosis prevalence.

## Abbreviations

HR	Hazard ratio
HPFS	Health Professionals Follow-up Study
*I* ^2^	Percentage of the variability in effect estimates that is due to heterogeneity
IPD	Individual participant data
LOO	Leave-one-out (influence) analysis
NHANES III	Third National Health and Nutrition Examination Survey
NOS	Newcastle–Ottawa Scale
NNT	Number needed to treat
OR	Odds ratio
PAF	Population attributable fraction
PR	Prevalence ratio
PRISMA	Preferred Reporting Items for Systematic Reviews and Meta-Analyses
PROSPERO	International Prospective Register of Systematic Reviews
RCT	Randomised controlled trial
RIS	Required information size
RoB	Risk of Bias
ROBINS-I	Risk Of Bias In Non-randomized Studies—of Interventions
RR	Relative risk
TSA	Trial sequential analysis
α	Probability of a Type I error
β	Probability of a Type II error
k	Number of studies
τ^2^	Between-study variance in random-effects meta-analysis

## Appendix A

**Table A1:** Main studies included in this review.

#	First Author (Year)	Country	Design	Sample (*n*)	Nut Exposure	Outcome	Use in Analyses
1	Gear, J.S. (1979) [81]	UK	Cross-section	376	Lifelong vegetarian status (proxy for high nuts/seeds)	Prevalent diverticulosis	Reported narratively Prevalence meta-analysis RoB-weighted model Baujat plot
2	Strate, L.L. (2008) [47]	USA	Cohort (18 years)	47,228	Peanuts, walnuts, other nuts, popcorn (servings wk^−1^)	Incident diverticulitis	Reported narratively Core diverticulitis meta-analysis Dose–response analysis Threshold meta-analysis Tipping-point analysis E-value
3	Crowe, F.L. (2011) [48]	UK	Cohort (11.6 years)	~690,000	Total dietary fiber (nut component not isolated)	Hospitalized diverticular disease	Reported narratively (not meta-pooled)
4	Crowe, F.L. (2014) [52]	UK	Cohort (6 years)	690,075	Change in fiber intake including nuts	Hospitalized diverticular disease	Reported narratively (not meta-pooled)
5	Strate, L.L. (2017) [67]	USA	Cohort 26 years	46,295	“Prudent” dietary-pattern score (rich in nuts)	Incident diverticulitis	Reported narratively Sensitivity meta-analysis (only labelled to avoid double-counting)
6	Mahmood, W. (2018) [54]	Nordic	Cohort (7 years)	~80,000	Fruit-/veg-fiber (includes nut residues)	Hospital diverticular disease	Narrative only (not meta-pooled)
7	Lim, Y.K. (2020) [83]	Korea	Cross-section	3864	“Snack” dietary-pattern factor (contains nuts)	Right-colonic diverticulosis	Reported narratively Prevalence meta-analysis RoB-weighted model Baujat plot
8	Liu, Y.-H. (2021) [82]	Taiwan	Cross-section	5586	Betel-nut (areca) chewing (freq. categories)	Any diverticulosis	Reported narratively Prevalence meta-analysis RoB-weighted model Baujat plot
9	Barlowe, T. (2025) [66]	USA	Cohort (6 years)	29,916	Total nuts + seeds (quartiles, g d^−1^)	Incident diverticulitis	Reported narratively Core diverticulitis meta-analysis Prevalence meta-analysis Dose–response analysis Threshold meta-analysis Tipping-point analysis RoB-weighted model Baujat plot E-value
