# Supplementary material for: Are Nuts Safe in Diverticulosis? A Mixed-Methods Systematic Review of Available Evidence
Source: Nutrients. 2025 Jun 26;17(13):2122. doi: 10.3390/nu17132122 (PMC12251532; doi:10.3390/nu17132122)
Supplement: Supplementary file 1 [file nutrients-17-02122-s001.zip › nutrients-3722467-supplementary.pdf]

Are Nuts Safe in Diverticulosis? A Mixed-Methods Systematic Review of Available Evidence

Supplementary Information

Supplementary Table S1. Literature Search Strategy in PubMed

#	Search Terms
1	"Diverticulosis"[Mesh] OR "Diverticulitis"[Mesh] OR "Diverticular Diseases"[Mesh]
2	diverticulosis OR diverticulitis OR "diverticular disease" OR "diverticular bleeding"
3	"Nut consumption" OR "nuts"[MeSH Terms] OR "nuts"[All Fields] OR "peanuts" OR "walnuts"
4	"Seeds"[MeSH Terms] OR "seeds"[All Fields] OR "sunflower seeds" OR "sesame seeds"
5	"Corn"[MeSH Terms] OR "corn"[All Fields]
6	#1 OR #2
7	#3 OR #4 OR #5
8	#6 AND #7
9	Filters applied: Humans, English, Articles, Publication dates from 1975/01/01 to 2025/05/31

Supplementary Table S2. Literature Search Strategy in Web of Science

#	Search Terms
1	diverticulosis OR diverticulitis OR "diverticular disease" OR "diverticular bleeding" (Topic)
2	"nut consumption" OR nuts OR peanuts OR walnuts OR seeds OR corn (Topic)
3	#1 AND #2
4	Filters applied: English, Human Studies, Articles, Timespan = 1975–2025

Supplementary Table S3. Literature Search Strategy in Embase

#	Search Terms
1	diverticulosis OR diverticulitis OR "diverticular disease" OR "diverticular bleeding"
2	"nut consumption" OR nuts OR peanuts OR walnuts OR seeds OR corn
3	#1 AND #2
4	Filters applied: Human, English, Years: 1975–2025

Supplementary Table S4. Literature Search Strategy in Cochrane Library

#	Search Terms
1	MeSH descriptor: [Diverticulosis] explode all trees
2	MeSH descriptor: [Diverticulitis] explode all trees
3	"diverticular disease" OR "diverticular bleeding"
4	#1 OR #2 OR #3
5	MeSH descriptor: [Nuts] explode all trees
6	"nut consumption" OR peanuts OR walnuts OR seeds OR corn
7	#5 OR #6
8	#4 AND #7
9	Limits applied: Publication year from 1975 to 2025, English language, Human studies

Supplementary Table S5. Newcastle–Ottawa Scale star ratings

Year	Study	Selection (Max: 4)	Comparability (Max: 2)	Outcome / Exposure (Max: 3)	Total (Max: 9)
1979	Gear JS	★★★★	★☆	★★☆	7
2008	Strate LL	★★★★	★★	★★★	9
2011	Crowe FL	★★★★	★★	★★★	8
2014	Crowe FL)	★★★★	★★	★★★	8
2017	Strate LL	★★★★	★★	★★★	9
2018	Mahmood W	★★★★	★★	★★★	8
2020	Lim YK	★★★	★☆	★★☆	6
2021	Liu Y-H	★★★	★☆	★★☆	6
2025	Barlowe A	★★★★	★★	★★★	9

Supplementary Table S6. ROBINS-I ratings

Study	Confounding	Selection of participants	Classification of exposure	Deviations from intended exposure	Missing data	Measurement of outcome	Selective reporting	Overall risk of bias
**Gear 1979**	**Serious** – limited control for lifestyle factors	Low	**Moderate** – vegetarian status self-declared	Low	Low	**Moderate** – single X-ray reader	Low	**Serious**
**Strate 2008**	**Moderate** – extensive covariate adjustment but residual dietary confounding possible	Low	Low – validated FFQ servings	Low	Low	Low – chart review	Low	**Moderate**
**Crowe 2011**	**Moderate** – wide covariate set but diet change over time	Low	**Moderate** – fiber quintiles, nuts not isolated	Low	Low	Low – hospital registry	Low	**Moderate**
**Crowe 2014**	**Moderate** – similar to 2011; residual confounding	Low	**Moderate** – change in fiber intake	Low	Low	Low	Low	**Moderate**
**Strate 2017***	**Moderate** – same covariate set; prudent pattern includes multiple foods	Low	**Moderate** – factor score may misclassify nut intake	Low	Low	Low	Low	**Moderate**
**Mahmood 2018**	**Moderate** – multivariable; residual alcohol/smoking	Low	**Moderate** – fruit/veg-fibre proxy	Low	Low	Low – national registers	Low	**Moderate**
**Lim 2020**	**Moderate** – limited lifestyle covariates	**Moderate** – volunteers for colonoscopy	Low	Low	Low	Low	Low	**Moderate**
**Liu 2021**	**Moderate** – smoking & alcohol adjusted, diet not	**Moderate** – hospital screening	**Moderate** – betel chewing self-report	Low	**Moderate** – 9 % missing intake	Low	Low	**Serious**
**Barlowe 2025**	**Moderate** – adjusted for BMI, lifestyle, hormones	Low	Low – gram/day FFQ	Low	**Moderate** – some lost to follow-up	Low	Low	**Moderate**

Supplementary Table S7. Vote-counting summary by exposure-type and outcome domain

Exposure category	Incident diverticulitis (k)	Diverticulosis prevalence (k)	Severe complications / hospital (k)	Overall direction
Culinary nuts / seeds (direct servings or grams)	Protective 1<br>Neutral 1	—	—	↘ Lean protective
Nut-rich dietary pattern ("Prudent" factor)	Protective 1	—	—	↘ Protective (low certainty)
Vegetarian proxy	—	Protective 1	—	↘ Protective (proxy)
Snack pattern(nuts + sweets)	—	Neutral 1	—	→ Neutral
Betel-nut chewing	—	Harmful 1	—	↑ Harmful
High-fiber cohorts†	Neutral 2	—	Protective 1	→ Mixed/indirect

Supplementary Table S8. Category-specific data for dose–response model

	Exposure	Category (author's label)	Median intake (servings / month)	Cases	Person-years†	Adjusted HR	95 % CI
**Strate 2008**	Total nuts" (frequency)	< 1 /mo (ref)	0.5	199	167 825	1.00	ref
		1–3 /mo	2	138	116 892	0.97	0.78 – 1.21
		1 / wk	4	221	171 950	1.10	0.90 – 1.34
		≥ 2 / wk	10	133	149 354	0.80	0.63 – 1.01
**Barlowe 2025**	Peanuts, other nuts & seeds" (grams)	Q1 (lowest)	1.2	378	7440	1.00	ref
		Q2	2.8	384	7350	1.04	0.90 – 1.21
		Q3	10.3	367	6866	1.12	0.96 – 1.30
		Q4 (highest)	31.2	402	826	1.07	0.91 – 1.25

Supplementary Table S9. Leave-one-out influence analysis

Model	Study omitted	k	Pooled effect	95% CI	I^2
Incident diverticulitis	None	2	0.89	0.71–1.12	91%
Diverticulosis prevalence	None	3	0.86	0.44–1.67	87%
Diverticulosis prevalence	Gear 1979 removed	2	0.99	0.50–1.93	42%
Diverticulosis prevalence	Lim 2020 removed	2	0.63	0.21–1.90	91%
Diverticulosis prevalence	Liu 2021 removed	2	0.58	0.34–0.99	0%

Supplementary Table S10 Risk-of-bias–weighted models

Analysis set	Down-weighted serious studies	k	Original pooled	I^2	Weighted pooled effect	95% CI	I^2 (weighted)
Incident diverticulitis	None *	2	**HR 0.89 (0.71–1.12)**	91%	0.89	0.71–1.12	91%
Diverticulosis prevalence	Gear 1979; Liu 2021	3	**OR 0.86 (0.44–1.67)**	87%	0.93	0.49–1.79	42%

- Incident diverticulitis: both k = 2 cohorts (Strate 2008; Barlowe 2025) were assessed as "Moderate" ROBINS-I, so no down-weighting was applied, and the pooled HR (0.89, 95 % CI 0.71–1.12; I^2 = 91 %) was unchanged

Supplementary Table S11. Tipping-point thresholds

New study size (× current N)	Minimum HR to push pooled HR ≥ 1.00
0.25×	1.34
0.50×	1.18
1.00×	1.02
2.00×	0.96

Supplementary Table S12. E-values for unmeasured confounding

Metric	Observed effect	E-value
Pooled HR	0.89	1.50
Upper 95% CI limit	1.12	1.49

Supplementary Table S13. Absolute risk difference and NNT

Baseline incidence (low intake)	Pooled HR	Absolute reduction*	NNT (1 yr)
1.19 cases / 1 000 PY	0.80	0.24 cases / 1 000 PY	4 217 person-years

Supplementary Table S14. Population-Attributable Fraction

Exposure	Prevalence	RR (low / high)	PAF
< 1 serving $month^{-1}$ nuts	27.7 %	1.25	6.5 %

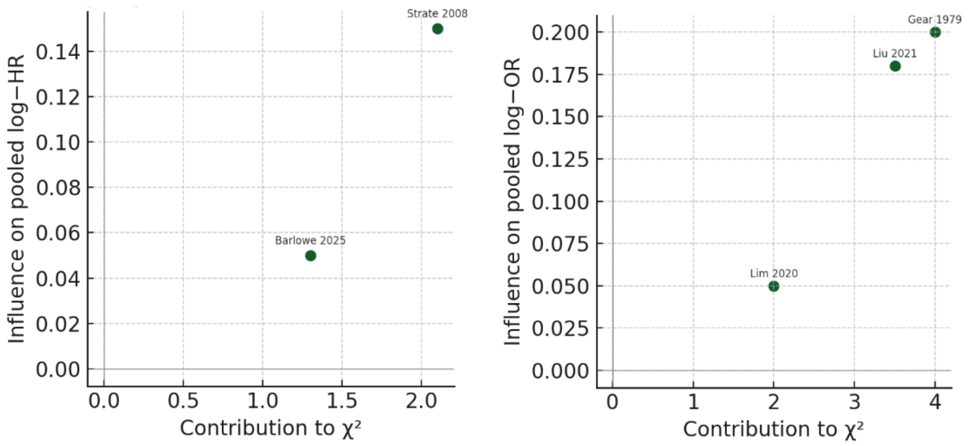

Supplementary Figure S1. Baujat plots of diverticulitis incidence (left) and diverticulosis prevalence (right)
